# Efficient generation of mice carrying homozygous double-floxp alleles using the Cas9-Avidin/Biotin-donor DNA system

**DOI:** 10.1038/cr.2017.29

**Published:** 2017-03-07

**Authors:** Ming Ma, Fengfeng Zhuang, Xiongbing Hu, Bolun Wang, Xian-Zi Wen, Jia-Fu Ji, Jianzhong Jeff Xi

**Affiliations:** 1 Department of Biomedical Engineering, State Key Laboratory of Natural and Biomimetic Drugs, College of Engineering, Peking University, Beijing, China; 2 Beijing Viewsolid Biotech Co. Ltd, Beijing 100071, China; 3 Division of Gastrointestinal Cancer Translational Research Laboratory, Department of Gastrointestinal Surgery, Key Laboratory of Carcinogenesis and Translational Research (Ministry of Education), Peking University Cancer Hospital & Institute, Fu-Cheng Road, Beijing, China; 4 State Key Laboratory of Biomembrane and Membrane Biotechnology, Institute of Molecular Medicine, Peking University, Beijing 100871, China; 5 Collaborative Innovation Center for Cardiovascular Disorders, Beijing Institute of Heart Lung and Blood Vessel Diseases, Beijing 100029, China

## Dear Editor,

The clustered, regularly interspaced, short palindromic repeats (CRISPR)/CRISPR-associated protein 9 (Cas9) system is a versatile tool for genomic engineering in mammalian cells and organisms, enabling the introduction of site-specific genomic double-strand breaks (DSBs)^1^. The resulting DSBs are repaired by at least two distinct and competitive mechanisms, nonhomologous end-joining (NHEJ) and homology-directed repair (HDR). The former results in insertions and deletions (indels), whereas the latter leads to precise genetic modification.

Precise genetic modification is of great utility in both research and clinical applications, even though the frequency of HDR is inherently low^2,3^. Several strategies have recently been developed to enhance the efficiency of HDR, including genetic or chemical interruption of the NHEJ pathway^4,5^, overexpression of a positive HDR regulator^6^ and manipulation of the cell cycle^7^. For example, Scr7, a DNA ligase IV inhibitor, has been shown to enhance HDR efficiency up to 2-4-fold in CRISPR/Cas9-manipulated embryos^4^. However, interference with endogenous genes has potentially detrimental side effects. Furthermore, such strategies are best suited to the knock-in of a short DNA fragment, normally no larger than 30 bp (Supplementary information, Figure S1A). The precise knock-in of a larger DNA fragment, such as one as large as 1 000 bp, is still a challenging task in mice^8^. Even for a donor DNA with ∼1-8 kb homology arms, the knock-in efficiency achieved by CRISPR/Cas9 was lower than 6% in mice^9,10,11,12^.

The Cas9 protein preferentially remains at the targeting site, where it first releases the non-target strand and could anchor there for as long as 6 h^13^. We hypothesized that the donor DNA searching for a homologous sequence across the entire genome is a key rate-limiting step of HDR, and thus the donor DNA would have a greater chance of being used if it is associated with CRISPR/Cas9 components. Here we demonstrate a simple and robust method of enhancing the HDR efficiency with no side effects detected. The newly developed system, termed the Cas9-Avidin-Biotin ssDNA (CAB) system, achieved an HDR frequency of ∼20% in mouse embryos for insertions of ∼1 kb.

**Figure 1:**
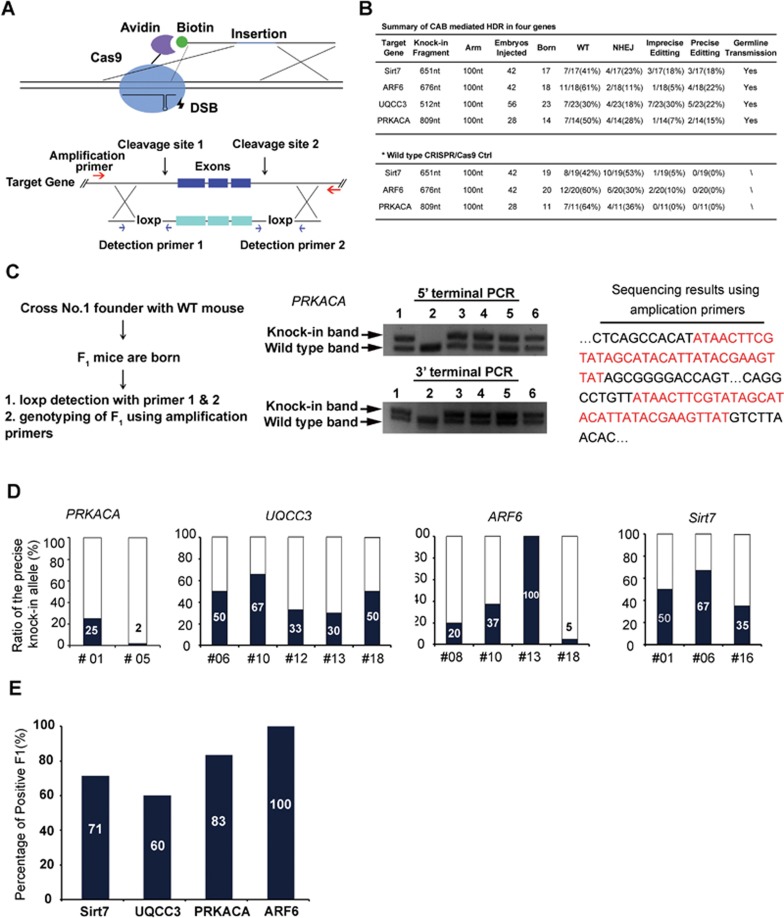
The CAB system enables precise insertion of double floxp sites with high efficiency. **(A)** Schematic diagram showing the CAB system. Above: a single-strand donor DNA is modified by biotin at the 5′-terminus and it contains an insertion sequence (blue line) flanked by homology arms. Guided by sgRNA, the Cas9-avidin fusion protein induces DSB at the desired locus and enriches the donor DNA. Bottom: strategy for inducing the double-floxp insertion in a targeted gene locus. A single-strand DNA donor contains two floxp sites, each with a homology arm of ∼100 nt at one side. Genome amplification primers (red arrows) are located outside the donor region. Two sets of loxp detection primers are located near the upstream/downstream floxp sites, respectively. Once a floxp site is successfully inserted, a larger band appears above the wild-type band, termed the knock-in band. **(B)** Summary of CAB-mediated HDR in four genes including *PRKACA*, *Sirt7*, *ARF6* and *UQCC3*. The control results using the wild-type CRISPR/Cas9 system are shown at the bottom. **(C)** Genome analyses of the F1 *PRKACA* mice. Left: scheme of the process and validation of germline transmission. Middle: upstream/downstream loxp insertion was detected using detection primer sets. Right: the complete sequence of the knock-in allele was analyzed by sequencing using amplification primers, and a representative sequence of a positive F1 is shown. **(D)** HDR-mediated knock-in of double floxp sites in the indicated loci. Genomic DNA was extracted from the tail of F0 pups and amplified by PCR using the detection primer sets to identify the loxp insertion. The ratio of the precise knock-in allele for each positive F0 pup was quantified according to the intensities of the knock-in band and wild-type band as shown in Supplementary information, Figure S2G-S2I. **(E)** Genotype analysis of F1 pups in all of the four genetic types of mice. In each type, one founder mouse, i.e., No. 6 in *Sirt7*, No. 10 in *UQCC3*, No. 1 in *PRKACA* and No. 13 in *ARF6*, was selected to cross with a wild-type mouse, and genomic DNA of F1 mice was then analyzed by PCR. Of note, all positive F1 mice carried one knock-in allele and one wild-type allele.

The CAB system is composed of a Cas9 fused with avidin via a flexible linker, an sgRNA and a biotin-modified single-strand donor DNA (biotin-ssDNA) (Figure 1A). Due to the high affinity between avidin and biotin, biotin-ssDNA would be enriched at the cleavage site, a hypothesis which was supported by the membrane labeling experiment (Supplementary information, Figure S1B). First, we optimized the CAB system, and found that Cas9 fused at its C-terminus to avidin still retains activity similar to wild-type Cas9 and the linker length should be of at least 16 amino acids (Supplementary information, Figure S1C). These results suggest that an intact Cas9 N-terminus is essential for nuclease activity, which is consistent with a previous structural analysis of Cas9^14^. Next, we assessed the performance of the optimized CAB system in 293T cells. The reporter assay showed a 2-fold enhancement of HDR efficiency, whereas sequencing analysis of editing at an endogenous gene locus revealed an elevation of the precise editing frequency by ∼5-fold (Supplementary information, Figure S1D-S1F). The CAB system also increased the ratio of the precise knock-in of a short DNA fragment up to 3-fold compared with the traditional CRISPR/Cas9 system in mouse zygotes (Supplementary information, Figure S2A-S2C).

The Cre/loxp system has been widely used to generate conditional knockout mice. Thus, we investigated whether the CAB system can achieve the knock-in of double floxp sites in one step. The CAB or CRISPR/Cas9 components were microinjected into a fertilized mouse zygote. The manipulated embryos were cultured to the two-cell stage and transferred into pseudopregnant females. The tails of the pups carried to term were harvested for PCR and sequencing analyses.

*PRKACA*, the gene for the catalytic subunit α of protein kinase A, has been linked to Cushing's syndrome, adrenocortical tumors, hepatocellular carcinoma and other diseases due to mutation or abnormal fusion. We sought to generate mice carrying two floxp sites inserted in the fifth and eighth introns of *PRKACA*, respectively (Supplementary information, Figure S2D). Two cleavage sites were selected according to the cutting efficiency of the CRISPR/Cas9 system, and a ∼1 000-nt donor DNA flanked by 100-nt homology arms was synthesized.

50% of the embryos manipulated by the CAB system generated live pups, demonstrating a survival rate comparable to that yielded by the CRISPR/Cas9 system (Figure 1B). At 7 days of age, DNA extracted from the pup tails was analyzed by PCR using two detection primer sets and sequencing using amplification primers. No precise editing was observed for the CRISPR/Cas9 system, and 36% of mice (4 out of 11) exhibited indels, indicating that DSBs were repaired via NHEJ (Figure 1B). In the CAB group, ∼15% pups (2 out of 14, i.e., No. 1 and No. 5) exhibited precise knock-in, which was confirmed by DNA sequencing (Figure 1B and Supplementary information, Figure S2E). The percentages of the precise knock-in alleles in the No. 1 and No. 5 mice were approximately 25% and 2%, respectively (Figure 1D). To demonstrate the successful germline transmission, the No. 1 founder mouse was crossed with a wild-type mouse, and five of six offspring were shown to carry the knock-in allele (Figure 1C and Supplementary information, Figure S2F).

To further demonstrate the robustness of the CAB system, we generated three additional mouse genetic models carrying double floxp sites at *UQCC3*, *ARF6* or *Sirt7* gene (Figure 1B and Supplementary information, Figure S2D). Genotyping performed 7 days after the pup birth showed that 18%-22% of the F0 mice carried the knock-in alleles (Figure 1B), suggesting that the improvement in precise editing obtained using the CAB system is not limited to a particular locus. It is noteworthy that a large proportion of the positive F0 pups exhibited a ratio of over 50% for the precise knock-in allele (Figure 1D and Supplementary information, Figure S2G-S2I). Particularly in case of the *ARF6* locus, the No. 13 F0 pup is likely homozygous for the knock-in allele as PCR analysis only revealed the knock-in band (Figure 1D and Supplementary information, Figure S2G) and all 14 TA clones sequenced were positive for double floxp sites (data not shown).

Finally, we selected one F0 founder from each genetic type and crossed them with wild-type mice for germline transmission analysis. All four F0 founder mice produced F1 pups carrying the knock-in allele. Of note, 60%-100% of the F1 pups of each genotype harbored the knock-in allele (Figure 1E), suggesting that a large proportion of germ cells of the four F0 founders carried the knock-in allele. Thus, it is likely feasible to generate a heterozygous conditional knockout mouse within 6 months after obtaining the F0 founders by crossing the F0 mice harboring high levels of knock-in mosaicism with a Cre mouse.

Here we report a simple and robust system to enhance HDR-mediated precise genomic editing in mouse embryos via the enrichment of donor DNA at the cleavage sites. Over 5 000 genetic diseases exist in humans; however, less than 10% have been mechanistically characterized and no gene editing therapy has been approved by FDA for clinical use. CRISPR/Cas9 is a powerful tool for gene modification in a variety of organisms as well as human cells^1^. A large number of challenging issues need to be addressed before this powerful technology can come into clinical use, including the low efficiency of gene correction based on HDR. Using the CAB system, we have improved the HDR efficiency such that HDR-based precise editing occurred in ∼20% of the manipulated embryos.

In summary, this system has three advantages. First, the donor DNA requires only two short homology arms of < 100 nt and thus can be easily synthesized. This is in contrast with traditional CRISPR/Cas9-mediated precise editing which requires long homology arms of a few kbs^9,10,11,12^. Second, this system does not cause additional toxicity compared with the CRISPR/Cas9 system, as evidenced by a similar survival rate of the F0 pups in embryos manipulated by these two systems. Safety is a critical concern for any CRISPR system in terms of future clinical application. Although chemical or genetic interruption of the NHEJ pathway can favor HDR, such manipulation may have a detrimental effect on embryonic development. It has been reported that Scr7 treatment results in defects in lymphocyte development^15^. Finally, we observed that 60%-100% of the F1 pups carried the knock-in allele with double floxp sites, suggesting that the new system would enhance the efficiency of generating a mouse model with precise genetic modification and thus save time compared with conventional approaches.

## Supplementary Information

Supplementary information, Figure S1 Construction and optimization of the CAB system.

Supplementary information, Figure S2 The CAB system increased the HDR ratio in mouse zygotes and generation of mice having double floxp sites in the four genetic types of mice.

Supplementary information, Table S1 Sequences and Primers

Supplementary information, Data S1 Extended discussion, Materials and Methods
