# Supplementary material for: Efficient generation of mice carrying homozygous double-floxp alleles using the Cas9-Avidin/Biotin-donor DNA system
Source: Cell Res. 2017 Mar 7;27(4):578–81. doi: 10.1038/cr.2017.29 (PMC5385615; doi:10.1038/cr.2017.29)
Supplement: Supplementary information, Figure S1 — Construction and optimization of the CAB system. [file cr201729x1.pdf]

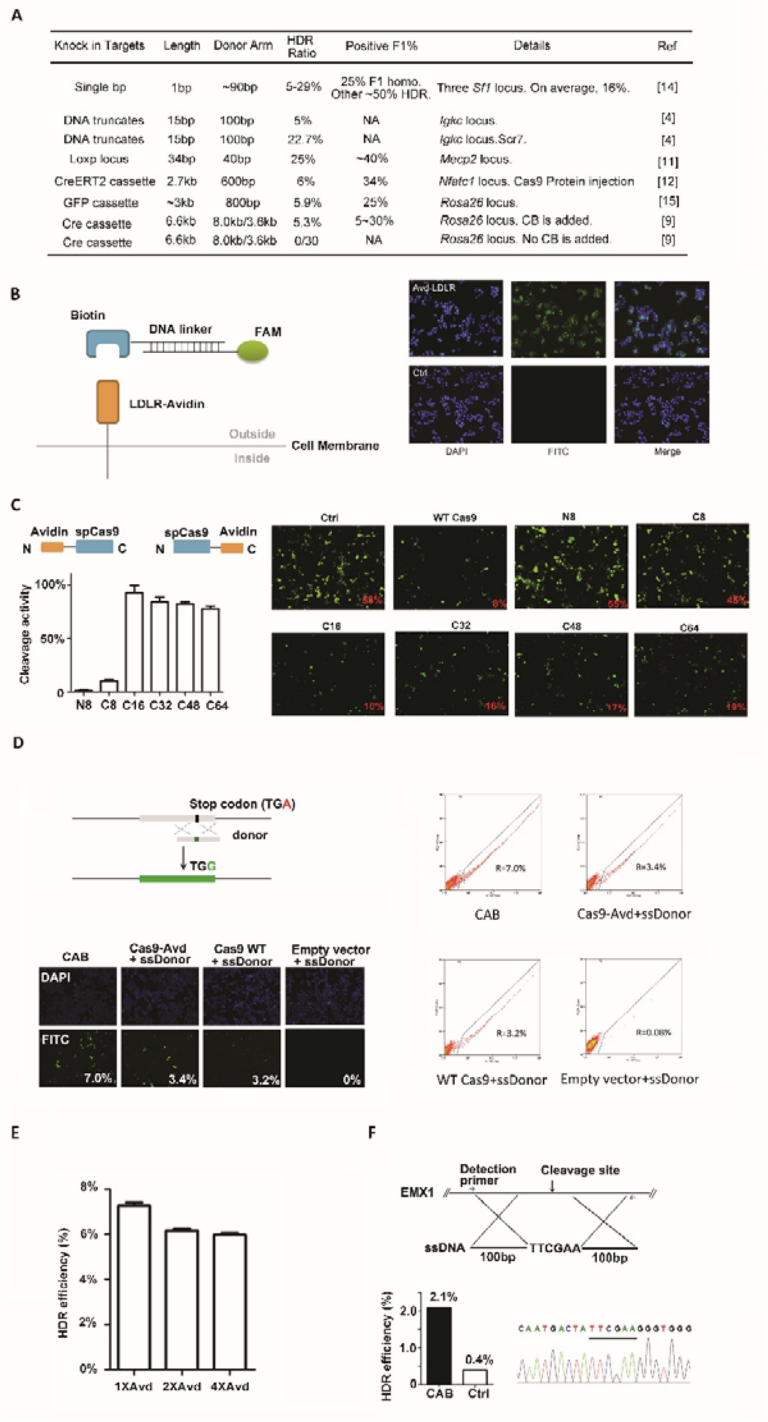

Knock in Targets	Length	Donor Arm	HDR Ratio	Positive F1%	Details	Ref
Single bp	1bp	~90bp	5-29%	25% F1 homo. Other ~50% HDR.	Three *Sf1* locus. On average, 16%.	[14]
DNA truncates	15bp	100bp	5%	NA	*Igkc* locus.	[4]
DNA truncates	15bp	100bp	22.7%	NA	*Igkc* locus.Scr7.	[4]
Loxp locus	34bp	40bp	25%	~40%	*Mecp2* locus.	[11]
CreERT2 cassette	2.7kb	600bp	6%	34%	*Nfatc1* locus. Cas9 Protein injection	[12]
GFP cassette	~3kb	800bp	5.9%	25%	*Rosa26* locus.	[15]
Cre cassette	6.6kb	8.0kb/3.6kb	5.3%	5~30%	*Rosa26* locus. CB is added.	[9]
Cre cassette	6.6kb	8.0kb/3.6kb	0/30	NA	*Rosa26* locus. No CB is added.	[9]

Supplement information Figure S1 Construction and optimization of the CAB system.

A) Summary of previous methods for gene knock-in in mouse zygotes.

B) Membrane labeling experiment. When avidin is fused to the outside terminal of Low-Density Lipoprotein Receptor (LDLR), a cell-surface protein and was over-expressed in 293T cells, a duplex DNA modified by biotin and FAM would form a green circle around the cells. Otherwise, no green

circle was observed.

C) Optimization of the Cas9-avidin protein in order to maintain its nuclease activity. Left: Avidin was fused to either the N terminal or C terminal of Cas9 via a linker with a length of 8 to 64 amino acids. N8 indicates avidin fused at the N terminal of Cas9 with an 8 amino acid linker, and so on. The nuclease activity of each fusion protein was normalized by the wild type Cas9. Right: fusion proteins were tested in a nuclease activity detection assay. A low dosage of Venus over-expressing plasmids mixed with spCas9/sgRNA plasmids were transfected into 293T cells. The sgRNA plasmid targeted the core florescence region of Venus, thus reducing the florescence signal. The percentages of green cells were calculated by normalizing them with the total cell numbers. The control group contained an empty vector instead of spCas9 plasmid.

D) The CAB system enhanced the HDR efficiency over 2-fold in a Venus reporter system in 293T cells. A stable 293T cell line was expressed with a Venus reporter that has a stop codon in the middle, thus generating no fluorescent signal. Once HDR had occurred, the stop codon was rescued and the reporter displayed green florescence. The percentage of positive green cells was calculated by normalizing the total cell number and FACS. The donor size is 81nt.

E) More avidins were fused with spCas9. A stable 293T cell line was expressed with a Venus reporter that has a stop codon in the middle, thus generating no fluorescent signal. Once HDR had taken place, the stop codon was corrected and the reporter displayed green florescence. The percentage of positive green cells was calculated by normalization with the total cell number.

F) The CAB system enhanced the HDR efficiency over 5-fold at an endogenous gene. The donor size is 206 nt, a 6 nt DNA truncated sequence flanked by two 100 nt homology arms. Genome detection primers were located outside the region of the donor, as indicated by the blue arrows. The HDR efficiency was quantified on the basis of the clone sequence as shown below. A representative sequence result is shown in the right corner, with the insertion underlined. For the biotin group, the positive TA clone number is 4 out of 192. For the control group, positive TA clone number is 1 out of 256.
