# Supplementary material for: Efficient generation of mice carrying homozygous double-floxp alleles using the Cas9-Avidin/Biotin-donor DNA system
Source: Cell Res. 2017 Mar 7;27(4):578–81. doi: 10.1038/cr.2017.29 (PMC5385615; doi:10.1038/cr.2017.29)
Supplement: Supplementary information, Figure S2 — The CAB system increased the HDR ratio in mouse zygotes and generation of mice having double floxp sites in the four genetic types of mice. [file cr201729x2.pdf]

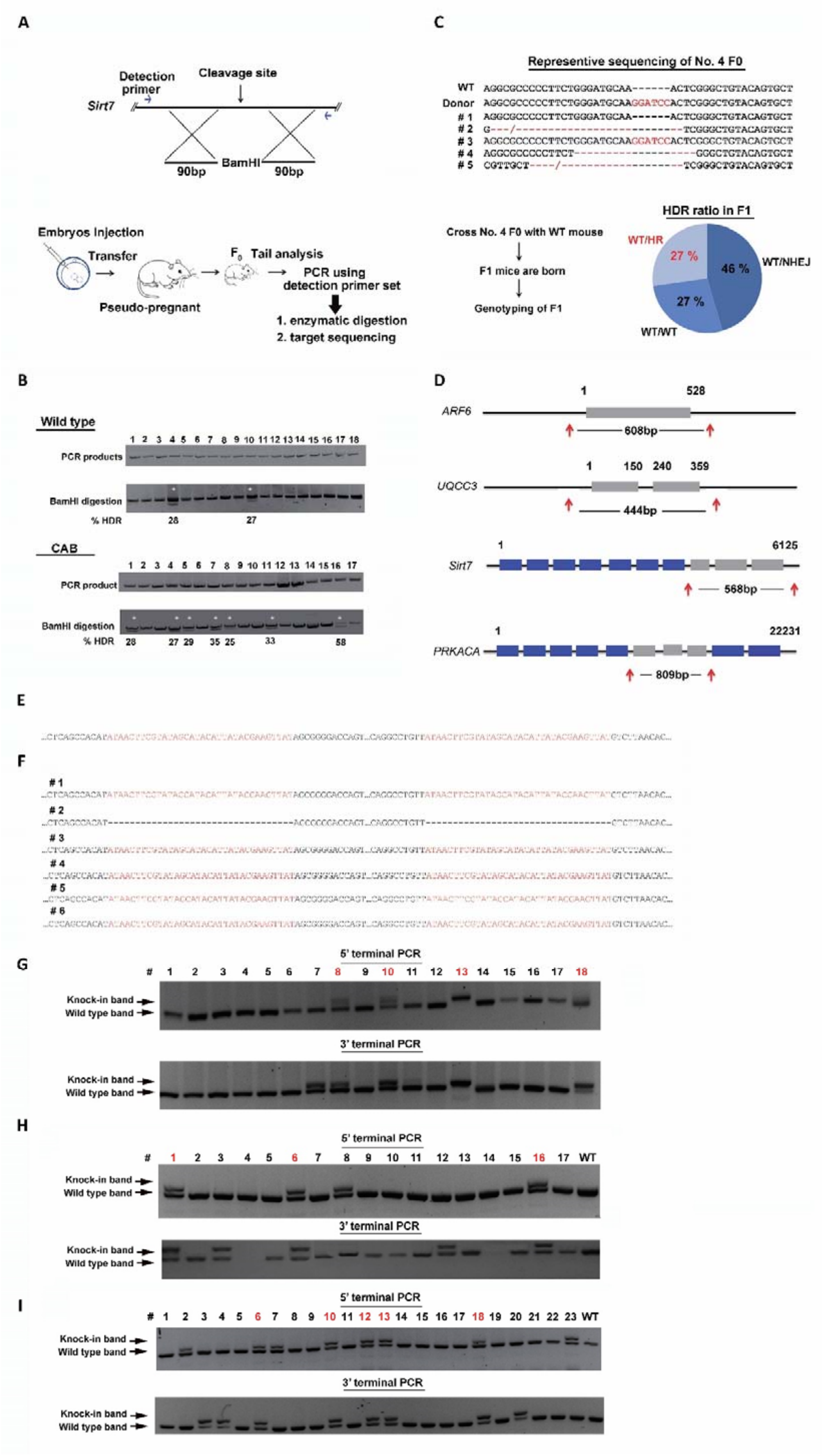
A
Detection primer
Cleavage site
Sirt7
90bp
BamHI
90bp
Embryos injection
Transfer
Pseudo-pregnant
F0 Tail analysis
PCR using detection primer set
1. enzymatic digestion
2. target sequencing
C
Representive sequencing of No. 4 F0
WT AGGCGCCCCCTTCTGGGATGCAA------ACTCGGGCTGTACAGTGCT
Donor AGGCGCCCCCTTCTGGGATGCAAGGATCCACTCGGGCTGTACAGTGCT
Cross No. 4 F0 with WT mouse
F1 mice are born
Genotyping of F1
HDR ratio in F1
WT/HR 27 %
46 % WT/NHEJ
27 % WT/WT
B
Wild type
PCR products
BamHI digestion
% HDR 28 27
CAB
PCR product
BamHI digestion
% HDR 28 27 29 35 25 33 58
D
ARF6 1 528 608bp
UQCC3 1 150 240 359 444bp
Sirt7 1 6125 568bp
PRKACA 1 22231 809bp
E
F
G
5' terminal PCR
Knock-in band
Wild type band
3' terminal PCR
Knock-in band
Wild type band
H
5' terminal PCR
Knock-in band
Wild type band
3' terminal PCR
Knock-in band
Wild type band
I
5' terminal PCR
Knock-in band
Wild type band
3' terminal PCR
Knock-in band
Wild type band

Supplementary information, Figure S2 The CAB system increased the HDR ratio in mouse zygotes and generation of mice having double floxp sites in the four genetic types of mice.

A) Schematic of the cleavage site and the template containing the *BamHI* at *Sirt7* locus in a mouse (Above). A single strand donor was synthesized that contained a *BamHI* locus flanked by two 90 nt homology arms. The detection primer set was located outside the donor region, as indicated by the blue arrows. Scheme of the production and characterization of the knock-in mouse (bottom) was shown. A mixture of Cas9-Avidin mRNA, sgRNA and the Biotin-donor were injected into fertilized eggs, and then the eggs were transferred to a Pseudo-pregnant mother mouse. After the baby mice were born (F0), the genome was extracted from their tails and analyzed by enzymatic digestion (1) or target sequencing (2).

B) The enzymatic digestion assays. Wild type: a wild type Cas9/sgRNA mRNA and a non-modified donor; CAB: Cas9-avidin/sgRNA mRNA and a biotin-ssDNA. The complete PCR product was 840bp, and a successful HDR sample contained a *BamHI* locus and produced a minor 740bp band upon enzymatic digestion, which is marked as a star in the gel. The efficiency of HDR was calculated on the basis of the density of the minor band in comparison with the main band.

C) Genotype analysis of No. 4 F0 and F1. A representative sequencing result of the No. 4 founder mouse is shown above. A founder mouse was crossed with a wild type mouse, and F1 mice were then genotyped. The ratio of the precise knock-in allele occurred at 27%, as shown in the pie chart.

D) Details of double floxp knock-in design on four targeted genes. Two same-direction floxps are inserted into intron regions, as shown in red arrows. Boxes represent exons of genes and solid lines represent introns. The length of knock-in truncates is highlighted below.

E) Example sequence of positive F0 carrying precise double floxp knock-in in the *PRKACA* locus. Using genome amplification primer, genome sequences were amplified and ligated into T vector for sequencing validation.

F) Crossing of the No.1 *PRKACA* double floxp knock-in mouse with a wild type mouse, 5 F1 pups were born. Genotyping of F1 was performed, and a floxp sequence upstream/downstream was

highlighted in red.

G) Gel detection of the *ARF6* locus upstream and downstream loxp insertion using detection primer sets. Genome DNA was extracted from the tail of F0 pups and amplified by PCR using the detection primer sets to identify the loxp insertion in the *ARF6* locus upstream and downstream. The knock-in band and wild type band are marked by arrows.

H) Gel detection of the *Sirt7* locus upstream and downstream loxp insertion sites using detection primer sets. Genome DNA was extracted from the tail of F0 pups and amplified by PCR using the detection primer sets to identify the loxp insertion in the *Sirt7* locus upstream and downstream. The knock-in band and wild type band are marked.

I) Gel detection of the *UQCC3* locus upstream and downstream loxp insertion sites using detection primer sets. Genome DNA was extracted from the tail of F0 pups and amplified by PCR using the detection primer sets to identify the loxp insertion in the *UQCC3* locus upstream and downstream. The knock-in band and wild type band are marked.
