# Supplementary material for: Efficient generation of mice carrying homozygous double-floxp alleles using the Cas9-Avidin/Biotin-donor DNA system
Source: Cell Res. 2017 Mar 7;27(4):578–81. doi: 10.1038/cr.2017.29 (PMC5385615; doi:10.1038/cr.2017.29)
Supplement: Supplementary information, Table S1 — Sequences and Primers [file cr201729x3.pdf]

Supplementary information, Table S1 Sequences and Primers

**Name**	**Sequences (from 5' to 3')**
EMX-F	TTCCTGCCCTGCCATCCCCTTC
EMX-R	AGCCAGCCCATTGCTTGTCCCT
EMX-donor	GAAGAAGAAGGGCTCCCATCACATCAACCGGTGGCGCATTGCCACGAAGCAG<br>GCCAATGGGGAGGACATCGATGTCACCTCCAATGACTATTCGAAGGGTGGGC<br>AACCACAAACCCACGAGGGCAGAGTGCTGCTTGCTGCTGGCCAGGCCCCTGC<br>GTGGGCCCAAGCTGGACTCTGGCCACTCCC
sgRNA scaffold	TAATACGACTCACTATAGGTCACCTCCAATGACTAGGGGTTTAAGAGCTATGC<br>TGGAAACAGCATAGCAAGTTTAAATAAGGCTAGTCCGTTATCAACTTGAAAA<br>AGTGGCACCGAGTCGGTGCTTTTTTT
***Sirt7*-BamHI knock in**	
Sirt7-genome-F	TGTATATGATCAATAAACGCCT
Sirt7-genome-R	GGAATGTGCCACCACTGTCACGTTG
Sirt7-sgRNA target	CCTTCTGGGATGCAAACTCGG
Sirt7-donor	CTTGGTTGTCTACACGGGCGCTGGAATCAGCACAGTGAGTGAGGGCCTGCGC<br>GTTGCTTCGGGTGTGAGGCGCCCCCTTCTGGGATGCAAGGATCCACTCGGGCT<br>GTACAGTGCTGGAGTTCAAAATCTCGCTCACCTTTGAAGTAACTTCAAGGTGT<br>CCTTTCTCAGAGACCCGAAGCAATTCTA
***PRKACA* double floxp knock in**	
PRKACA-genome-F	TTGACACAGGGTCTCACTTTGTAGTCTG
PRKACA-genome-R	TCTTTAGTTGAGCCATCAACCCCTG
PRKACA-sgRNA up	CTCAGCCTCAGCCACATAGCGGG
PRKACA-sgRNA down	GGCTCCTGTGTTAAGACAACAGG
PRKACA-donor	TATGTCAGGCTCCCAAGTTTTTACAATAGTTTGGGTCTCTGTCAGGCTGCCCTT<br>GGAATCTTGGACTCATGCAGTCCCCCTGCCTCAGCCTCAGCCACATATAACTT<br>CGTATAGCATACATTATACGAAGTTATAGCGGGGACCAGTGGTACATGGCAT<br>CCCCAGCCTTTAGTCTACATTTGATAAGCTAGACACGGGGTGTCTTGCTCTGT<br>GGGTACACAGACCCACATGCTGGACCACTTGGGGATGTGGTCATATTCACCC<br>ACACATCCCCTCCCGGCTTGTCCCACCAGCGAGCCCCATGCCCGTTTCTACGC<br>GGCGCAGATCGTCCTGACCTTTGAGTATCTGCACTCCCTGGACCTCATCTACC<br>GGGACCTGAAGCCCGAGAATCTTCTCATCGACCAGCAGGGCTATATTCAGGT<br>GCCCGAGGCCGGGGGAGGGCACTCGAGGGCACATTTGGAGCCTGCAGCCCTT

	CTCTCTACCAACTGCTCATTCTTGTGCCTACAGGTGACAGACTTCGGTTTTGCC<br>AAGCGTGTGAAAGGCCGTACTTGGACCTTGTGTGGGACCCCTGAGTACTTGG<br>CCCCCGAGATTATCCTGAGCAAAGTAGGCACCTCAACCAGCCTGCCCCACCC<br>CTGAGGCCTACTCTACCTCACTAGCCCGCCCCACCCCTGAGGAATCACCTCCC<br>TCTTCACCTTGCCTCATCGAGTGGCCCCCCCATCTTGCTCTAGGGCTACAACA<br>AGGCTGTGGACTGGTGGGCTCTCGGAGTCCTCATCTACGAGATGGCTGCTGGT<br>TACCCACCCTTCTTCGCTGACCAGCCTATCCAGATCTATGAGAAAATCGTCTC<br>TGGGAAGGTGAGGCCAGGATACGGATTTCAGCTCTGGAAGGAATCAAAACA<br>GCCTATCACATGTCCTCACAAGGCTGAGTATGCTGTCACAGGCCTGTTATAAC<br>TTCGTATAGCATACATTATACGAAGTTATGTCTTAACACAGGAGCCTGAGGCA<br>GAAGGATCTTAAGTTTGAAGCCAGCATGGGCTACATAGCGATTTCCAGGATA<br>GCATGGGCTGAGAAAATAGTAAAA
PRKACA 5’ floxp-F	ATGTCCTACTATGTCAGGCTCC
PRKACA 5’ floxp-R	TCAAATGTAGACTAAAGGCTGGG
PRKACA 3’ floxp-F	ACGGATTTCAGCTCTGGAAGGA
PRKACA 3’ floxp-R	TTAGTTGAGCCATCAACCCCTGTC
***ARF6* double floxp knock in**	
ARF6-genome-F	TTGCTCGCTCGCTCGTTCGCG
ARF6-genome-R	AAGCCATCTACAGCAAGTGATA
ARF6-up target	CGGGACACCTGAATGCCCCCGG
ARF6-down target	GGAGGTGGCGATAATCCTAGG
ARF6-donor	CAGCTTCGCGCAGGCCGCAGGGCGTCCTCGCAGCGGGGCGGCGACGTTTCGG<br>GCTCGCGGCGGCGTTGTAGGCTTGAGGGGACCCGGGACACCTGAATGCATAA<br>CTTCGTATAGCATACATTATACGAAGTTATCCCCGGCCCCGGCTCTTCCGACG<br>CGATGGGGAAGGTGCTATCCAAGATCTTCGGGAACAAGGAAATGCGGATCCT<br>CATGCTGGGCCTGGACGCAGCCGGCAAGACAACGATCCTGTACAAGTTGAAG<br>CTGGGCCAATCGGTGACCACCATCCCCACGGTGGGCTTCAACGTGGAGACGG<br>TGACTTACAAAAACGTCAAGTTCAACGTGTGGGATGTGGGCGGCCAGGACAA<br>GATCCGGCCGCTCTGGCGGCATTACTACACCGGGACCCAGGGTCTGATCTTCG<br>TGGTAGACTGCGCCGACCGCGACCGCATCGACGAGGCCCGCCAGGAGCTGCA<br>CCGCATTATCAATGACCGGGAGATGAGGGACGCCATCATCCTCATCTTCGCC<br>AACAAGCAGGACCTGCCCGATGCCATGAAACCCCATGAGATCCAGGAGAAA<br>CTGGGCCTGACCCGGATTCGGGACAGGAACTGGTATGTGCAGCCCTCCTGTG<br>CCACCTCCGGGGACGGACTCTATGAGGGGCTCACATGGTTAACCTCTAACTA

	CAAATCCTAATGAGCGTCCTCCACCCAGCCCCCGGAAGGAGAGAAATCCAAA ACCCATTCCTAGGATAACTTCGTATAGCATACATTATACGAAGTTATATTATC GCCACCTCCATCACCTCTTTGAATTGCCACTCTCTTTTTGAATCTGAACTCTGG AGTTACTGTTCTACAGTTTAGTGGGGTTGGGGGTTTTCTT
ARF6 5’floxp-F	AGCTTCGCGCAGGCCGCAGGG
ARF6 5’floxp-R	ATGAGGATCCGCATTTCCTTG
ARF6 3’floxp-F	TCCTAATGAGCGTCCTCCACCCA
ARF6-3’loxp-R	AACTGTAGAACAGTAACTCCAGAG
***Sirt7* double floxp knock in**	
Sirt7-genome-F	TGTATATGATCAATAAACGCCT
Sirt7-genome-R	GGAATGTGCCACCACTGTCACGTTG
Sirt7-up target	CTGTAGCTGCCGGATGAGG<u>CGG</u>
Sirt7-down target	CCTTCTGGGATGCAAACT<u>CGG</u>
Sirt7-donor	GGTATTTCTTTGAAATCCCGAAGGAGCAAGGGTGGGAAGGGCGAGCCGGAA GAGGTGGAAAGGGCCGGGCCCGCGCATGCGTCTCTGTAGCTGCCGGATGATA ACTTCGTATAGCATACATTATACGAAGTTATAGGCGGAAGCGGAAGCCGGAA GCGCAGTCAAAGGAGCGATGGCAGCCGGTGGCGGTCTGAGCCGCTCGGAGC GCAAAGCTGCTGAGCGGGTCCGGAGGCTGCGGGAGGAGCAGCAGCGGGAGC GCCTCCGCCAGGTGAGCCACTGCCGTGCCCGCGCTCGCGCCGGCGCGCGCGG TCGCTCACCCGCTGCTCGTCCGTAGGTGTCACGCATCCTGAGGAAGGCGGCTG CAGAGCGCAGCGCGGAGGAGGGCCGGCTCTTGGCCGAGAGCGAGGATCTGG TGACCGAGCTGCAGGGTCGAAGTCGGCGGCGTGAGGGCCTCAAGCGCCGCCA GGAGGAGGCGAGTCGCGGGCAGCGGGTGCTGGGCGGCGGGCTGCGGGAGGC GAGCGGCGGCAATAACCAGCCTGGCTCCTATCAGGTGTGTGATGACCCGGAG GAGCTGCGGAGGAAGGTCCGCGAACTGGCCGGAGCTGTCCGAAGTGCCAGG CACTTGGTTGTCTACACGGGCGCTGGAATCAGCACAGTGAGTGAGGGCCTGC GCGTTGCTTCGGGTGTGAGGCGCCCCCTTCTGGGATGCAAATAACTTCGTATA GCATACATTATACGAAGTTATACTCGGGCTGTACAGTGCTGGAGTTCAAAATC TCGCTCACCTTTGAAGTAACTTCAAGGTGTCCTTTCTCAGAGACCCGAAGCAA TTCTAGATTTAAGAC
Sirt7 5’floxp-F	AAGGGCGAGCCGGAAGAGGTGGA
Sirt7 5’floxp-R	GCCATCGCTCCTTTGACTGCGCTTC
Sirt7 3’floxp-F	GTGCCAGGCATTTGGTTGTCTAC
Sirt7 3’floxp-R	CGGGTCTCTGAGAAAGGACACC

***UQCC3* double floxp knock in**	
UQCC3-genome-F	GGATGTCTGTATGAGAGTGTCAGA
UQCC3-genome-R	GGTCTTCAGCGCACATTGCGCTC
UQCC3-up target	ATGTCATGGCTTCCTTCTAGG
UQCC3-down target	CCAAGAAAGGAGCCTTACAGG
UQCC3-donor	TCTCTAGCCCAGCTGTTTCTCTATGATTTGAGGCCCGGCCCCGCCCCTCTCCTA<br>GGTCGCTCTAGCCCGCATAGGGAGCGGTGAGATGTCATGGCTTCCTATAACTT<br>CGTATAGCATACATTATACGAAGTTATTCTAGGCTGCGGGAGGAAGCGGACG<br>GCACCTCTTTTGTAGTGTAATCCGCCATGGAGGTGGCTCGTAAAGCACTTGTG<br>GCAGTTGCAGTGCTAGGCGGGGGAGCTGGCGTGGGTTCTATTCTGTTTGCTCT<br>TGTGACCCCAGGAGAACTACAGAAGCAGTCGATGCTGCAGGTAAAAACAACC<br>GGACTCCCCGAGCTGGGTGTGCGTGGGACGAGCCCCTGTCTTTTCGCCCAGA<br>ATGCAAACATCCTTCTTGCCCGCAGGAGATGCCGGAAAGGGACTCGCGGCGC<br>AGGGACGAAGCAGTCAGGACCACGGAACTGGTGATGGCTACCCTGAAGGAC<br>GCCGCAGCCACGAAGGAGAACGTGGCCTGGAGGAGAAACTGGACAGTTAGC<br>GGGGATGGCAGGTCAGCATGACACCAGAGCTCGCCCGTGGTCACGGGAACCT<br>GTAATAACTTCGTATAGCATACATTATACGAAGTTATAGGCTCCTTTCTTGGT<br>GCTCCGGACATGGAGTCGGGAGATACACCAGGACTCTCTCCAAGTCACTGGG<br>AACTGCGGTTGGGGGAACGCCTTTAAAGAGCA
UQCC3 5’floxp-F	TTCTCTATGATTTGAGGCCCGGC
UQCC3 5’floxp-R	AACTGCCACAAGTGCTTTACGAG
UQCC3 3’floxp-F	GAGAACGTGGCCTGGAGGAGAAAC
UQCC3 3’floxp-R	ACCGCAGTTCCCAGTGACTTGGAG
