# Supplementary material for: Efficient generation of mice carrying homozygous double-floxp alleles using the Cas9-Avidin/Biotin-donor DNA system
Source: Cell Res. 2017 Mar 7;27(4):578–81. doi: 10.1038/cr.2017.29 (PMC5385615; doi:10.1038/cr.2017.29)
Supplement: Supplementary information, Data S1 — Extended discussion, Materials and Methods [file cr201729x4.pdf]

## **Supplementary information, Data S1** Extended discussion, Materials and Methods

### Construction and optimization of the CAB system in mammalian cells

A series of Cas9-Avidin fusion proteins were constructed with variable flexible linkers ranging from 8aa to 64aa, as shown in **Supplementary Figure 1C.** The cleavage activity of these fusion proteins was quantified by the fluorescent density observed in 293T cells in which GFP plasmids had been co-transfected with Cas9-Avidin variants as well as a targeted GFP sgRNA. The wild-type Cas9 demonstrated the cleavage efficiency of 86%. The Cas9 fused with avidin at the C terminal still remains activity similar to the wild type Cas9, and the linker length should be at least 16aa between Cas9 and avidin. These results show the N-terminal of Cas9 to be essential for the nuclease activity, a finding which is consistent with the previous structural analysis of Cas9 [1].

Next, we tested the HDR efficiency of the CAB system by using another reporter system **(Supplementary Figure 1D)**. In brief, a Venus reporter was constructed with a stop codon in the middle of coding sequence and incorporated into the genome of 293T cells, thus generating a stable cell line. This cell line produced no green fluorescence unless the stop codon was corrected by CAB-mediated HDR. The targeting template was comprised of the intended insert (i.e. correct codon) sandwiched between 100 nucleotide (nt) arms that were homologous to the sequence flanking the DSBs. The template was modified with biotin at the 5’ end and is termed the “biotin-donor”, or without biotin, the “control donor”. These donors were transfected into stable cells together with Cas9-Avidin/sgRNA plasmids or Cas9/sgRNA plasmids, respectively. The CAB system resulted in a more than 2-fold enhancement of HDR in comparison with the wild-type Cas9 system **(Supplementary Figure 1D)**. Furthermore, the addition of more avidins could not increase the HDR efficiency (**Supplementary Figure 1E**).

To examine the HDR efficiency of the CAB system in modifying an endogenous gene, a donor containing a “TTCGAA” fragment flanked with a 100bp arm on each side was designed to incorporate into the *EMX1* locus **(Supplementary Figure 1F)**. Three sgRNAs were synthesized, and then one was finally selected on the basis of its cleavage activity. After two days of transfection with the CAB system, target

sequences were cloned using detection primers and ligated into T-vectors for sequencing. The CAB system enhanced the HDR efficiency up to 5.3-fold.

**The CAB system enhanced the knock-in efficiency of a short truncated sequence in a mouse**

We then investigated the CAB-mediated HDR efficiency of short DNA fragments at a given locus in the fertilized mouse embryo. *Sirt7* plays a crucial role in chromatin remodeling, DNA damage and tumorigenicity. Moreover, previous studies showed that homozygous deletion of *Sirt7* resulted in a shorten lifespan, with some of animals exhibiting problems with embryonic development [2,3]. Thus, we designed a labeled *BamHI* restriction site at an intron locus of *Sirt7* that would be expected to exert a negligible effect on embryo development and survival (**Supplementary Figure 2A**). The CAB system and the wild type CRISPR system were respectively microinjected into fertilized zygotes. The manipulated embryos were cultured to the two-cell stage and transferred into pseudopregnant females. The tails of the pups carried to term were harvested for PCR using a detection primer set.

We first performed enzymatic digestion experiments. Forty one percent of baby mice (7/17) carried a *BamHI* locus at the target site in the groups using the CAB system, a 4-fold increase in comparison with the control group **(Supplementary Figure 2B)**. The ratio of knock-in allele was approximately 30% in all of the positive groups except for No. 16, in which 8 bases were somehow deleted upstream. The target sequences in the 9 positive groups were cloned into a T-vector and further confirmed by DNA sequencing. To demonstrate that the knock-in allele was stably transmitted from a founder to its offspring, the No. 4 founder mouse was crossed with a wild type mouse. Three F1 baby mice out of 11 carried the knock-in locus, that is, the knock-in efficiency reaching 27% **(Supplementary Figure 2C)**.

## Materials and Methods

### Cell Culture and Transfection

Cells are cultured in Dulbecco's Modified Eagle Medium (DMEM) containing 10% fetal bovine serum (FBS), 100 U/ml penicillin and 0.1 mg/ ml streptomycin (PS) under humidified conditions in 95% air and 5% $CO_2$ at 37 $^oC$.

Plasmids are transfected into cells using Lipo2000 (Invitrogen 11668019) for plasmids or CRISPR Max (Invitrogen CMAX 00015) for protein. PS were removed one day before plasmids or protein transfection. DMEM is replaced in 6 hours after transfection. Cas9-Avidin fusion protein is purified with His tag and sgRNA is transcribed *in vitro* using an In Vitro Transcription Kit (ViewSolid, VK-007). Single strand DNA donor is either ordered (for length <100bp) from Invitrogen or synthesized using single direction PCR (for length > 100bp). 24 hours after protein transfection, genomes are extracted from cells for further analysis.

### Plasmid Construction and Single Strand Donor Synthesis

The coding sequences for wild-type Cas9 (wtCas9) and Cas9-avidin fusion protein are constructed into a pcDNA3.1 plus vector. A targeted GFP sgRNA is also constructed into a pcDNA3.1 plus vector. The optimized linker sequence for Cas9-Avidin fusion protein is AGCGGTTCAGAGACCCCAGGAACTAGCGAGAGCGCTACACCGGAATCG. A Venus reporter is constructed by mutating TGG (W58) into a stop codon TGA. A single strand DNA donor is ordered from Invitrogen. Biotin is modified at 5' terminal. A Venus donor sequence (81nt) is: TGAAGTTCATCTGCACCACCGGCAAGCTGCCCGTGCCCTGGCCCACCCTCGTGACCACCCT GACCTACGGCGTGCAGTGC.

Single strand donor is synthesized using the Liner-After-The-Exponential (LATE)-PCR as described previously [4]. A biotin modified primer, which was ordered from Invitrogen, was used for the generation of the donor using the method of Liner-After-The-Exponential PCR. In brief, LATE-PCR reactions are carried out in a final volume of 25 μL with 100 nM forward primer and 1000 nM reverse primer modified with biotin by MightyAmp (Takara 074A).

## Cas9 Target Design and Optimization

Cas9 targets are designed according to the protocol described in the web of http://crispr.mit.edu. The cleavage efficiency is measured using *In Vitro* Detection Assay (ViewSolid VK-007). The genomic sequences are amplified and purified, and then used as cutting templates for each sgRNA. For each locus, four targets are designed and their efficiencies are measured, and then the most efficient target is selected.

## Cell Knock-in Analysis

The dosage of Cas9-Avidin fusion protein is 2 µg, sgRNA 100 ng, and ssDNA 100 ng for 50K cells. In 24 hours after transfection, cells are lysed at 55oC with Proteinase K for 30 minutes. Genomes are extracted using TianGen's Genome Extraction Kit (DP304). Target sequences are amplified using MightyAmp Kit (Takara 074A) and gel purified. After adding A at 3' terminal, they are ligated into T vector for sequencing to identify the correct knock-in efficiency.

## Zygote co-injection

All animal procedures are strictly performed according to Animal Care Guidelines. Fertilized zygotes are collected from ICR mouse oviducts. For injection, Cas9-Avidin mRNA (100 ng/µL), sgRNA (20 ng/µL) and ssDNA (20 ng/µL) are mixed, and then injected into zygotes. The injected zygotes are first cultured in KSOM with FBS at 37 $^{o}$C and 5% $CO_2$, and then transferred into pseudopregnant female ICR mice.

## Mouse Genotyping

Mouse tails are lysed at 55 $^{o}$C with Proteinase K overnight. Genomic DNA are extracted from F0 tails and amplified by MightyAmp (Takara 074A). After adding A at 3' terminal, they are ligased into a T vector to further sequencing. For floxp insertion detection, a small PCR target region is selected for both upstream and downstream loci. Since floxp inserted allele is 34 bp bigger than wild type allele, the knock-in efficiency could be calculated by comparing bands' florescence. The sequences for detection primers are listed in supplementary tables.

For founder mouse mosaicism detection, targeted genome region is amplified using related genome amplification primers. Desired DNA band is extracted from gel and then ligased into a T vector for further sequencing. The mosaicism ratio is calculated by the positive clone number against the whole clone number. Founder mouse mosaicism is also calculated using positive F1 carrying knock-in allele against the number of F1 infants.

**Founder Mouse Knock-in Efficiency Quantification**

The ratio of knock-in is calculated from positive TA clone ratio. First, tails of F0 mice are collected and genomes are extracted. Then, PCR amplifies targeted genes with upstream/downstream primers. This pair of primers locates out of donor region to avoid potential PCR contamination from donor. Third, targeted band is purified after gel electrophoresis and amplified by PCR using Taq enzyme and ligated into a T vector. Further sequencing is conducted on T vector. Finally, the ratio of knock-in is calculated from positive knock-in T vector normalized by the total T vector number.

**Reference:**


1. Hiroshi NF. Ann R, Patrick D. Hsu, *et al.*, Crystal structure of Cas9 in complex with guide RNA and target DNA. *Cell 2014*; **156:** 935-949.

2. Tatsuya Y, Md. FK, Yoshifumi S, *et al.*, SIRT7 controls hepatic lipid metabolism by regulating the ubiquitin-proteasome pathway. *Cell Metab 2014*; **19(4):** 712-21.

3. Olesya V, Christian S, Praveen G, *et al.*, Sirt7 increases stress resistance of cardiomyocytes and prevents apoptosis and inflammatory cardiomyopathy in mice. *Circ Res 2008*; **102(6):** 703-10.

4. Jesse JS, J.Anquiles S, Kenneth EP, *et al.*, Direct Amplification of Single-Stranded DNA for Pyrosequencing using Linear-After-The-Exponential (LATE)-PCR. *Anal Biochem 2006*; **353(1):** 124–132
